# Evidence-Based Laboratory Testing Algorithms: A Strategic Approach to Improving Diagnostic Accuracy

**DOI:** 10.7759/cureus.101862

**Published:** 2026-01-19

**Authors:** Kenneth N Okonkwo

**Affiliations:** 1 Department of Forensic Toxicology, Laboratory Corporation of America (Labcorp), Houston, USA

**Keywords:** accuracy, diagnostic approach, evidence-based medicine, laboratory medicine, testing algorithm

## Abstract

The increasing knowledge of evidence-based medicine (EBM) has reinforced the need for structured diagnostic approaches in laboratory testing. A laboratory testing algorithm - a stepwise, guideline-informed sequence of tests - can significantly improve diagnostic accuracy, reduce unnecessary testing, and boost clinical decision-making. These algorithms, combined with current evidence, clinical judgment, and test performance indices, offer clinicians a strategic framework for patient evaluation. In this review, we examine the concept, design, and application of evidence-based laboratory testing algorithms, with an example drawn from common clinical settings, such as the anemia testing algorithm.

The presentation of these pathways focuses on calling attention to how diagnostic efficiency can be optimized in daily clinical practice. The integration of these algorithms into electronic health records (EHR), computerized physician order entry (CPOE) systems, and clinical decision-support systems (CDSS) is also recommended. Despite the potential of these algorithms, limitations to widespread adoption still exist, which include a lack of standardization, provider familiarity, interpretive huddles, and system-level limitations. However, as healthcare systems continue to evolve and prioritize cost-effectiveness and patient safety, laboratory professionals have a distinctive opportunity to lead the development and implementation of testing algorithms.

This review also stresses the value of collaboration between laboratory professionals and clinical teams in shaping diagnostic strategies, and calls for expanded awareness, familiarization, education, and full integration of evidence-based approaches in laboratory medicine.

## Introduction and background

Patient care is a multi-disciplinary activity with a continued quest for a targeted and integrative approach to healthcare delivery, and at the center of this activity are the needs and values of those involved in both the delivery and the receiving of care [1]. In the care of patients, the combined knowledge and experience of the provider are crucial; hence, accurate diagnosis is essential in modern medicine, although this proficiency among physicians does not necessarily guarantee the desired treatment outcomes [2]. Clinical decision-making relies strongly on thoughtful skills that include natural ability and proficiency, but the training of healthcare professionals and making the best use of clinical interpretation through “physician calibration” are effective in lowering the risk of errors in diagnosis. Lippi and Cervellin defined physician calibration as a set of activities focused on defining the relationships between specific signs and symptoms present in a particular patient and the likelihood of a certain disease, supporting clinical decision-making with the most relevant and accurate diagnosis [3].

Diagnostic accuracy is paramount to the safety of patients, treatment guidance, and error prevention, because diagnostic errors remain widespread, thus contributing to damage that could otherwise be prevented, increased healthcare costs, and worsening disease conditions. Therefore, leveraging knowledge of diagnostic accuracy is essential for improving clinical outcomes [4,5]. Diagnosis is considered the bedrock for providing safe and effective medical care, and the ability of the physician to diagnose a patient’s illness is one of the distinctive features of medical expertise, fundamental to providing correct and effective treatment [6]. The diagnostic approach can be viewed as an active, multi-dimensional process of collecting and analyzing information, where data collection starts with a history and examination, which usually includes laboratory results, imaging, and invasive procedures. However, this process is laden with widespread errors, and the challenge becomes evident when one considers all the stages of making a correct diagnosis [7].

Now, therefore, the medical profession must consider how we can improve accuracy and timely diagnosis [8], because diagnostic accuracy relates to the capacity of a test to distinguish between the target condition and normal health [9]. Improving diagnostic accuracy is also tied to a physician’s ability to order the right tests to optimize laboratory test utilization, as incorrect utilization practices can lead to the overuse of some tests and the underuse of others [2]. Unnecessary laboratory testing is typically described as an area of low-value care, with significant healthcare quality and safety implications. Carrigan et al. suggest a reduction in low-value laboratory testing, such as serum folate, thrombophilia, and cancer screening markers, as these tests are identified as low yield, along with routine laboratory testing in hospitalized patients [10]. Over the years, low-value care has been a challenge, due to the overuse of tests and procedures in an inpatient setting [11], and overutilization of laboratory tests is now recognized as both harmful to patients and wasteful [12]. Diagnostic errors can originate from inappropriate test utilization, where practices are not in line with current expert knowledge or evidence-based guidelines for use [13].

Consequent upon these challenges, there is a need to focus on strategic approaches to improve diagnostic accuracy and diagnostic excellence in medical practice. This is where the clinical laboratory profession plays a role in promoting diagnostic accuracy by advocating for the use of laboratory testing algorithms. Diagnostic test utilization in the patient-care setting should be guided by evidence, yet many providers order tests without consideration of supporting evidence. Sensitivity and specificity are critical components of test accuracy, helping physicians gauge the suitability of a diagnostic tool [14].

## Review

Understanding evidence-based approaches and laboratory testing algorithms

*Evidence-Based Practice* (EBP)

From laboratory values to clinical decision-making is a practical, real-time display of translational healthcare in action, and so we will begin the discussion by looking at what an evidence-based approach entails, eventually narrowing down to evidence-based laboratory medicine (EBLM). Evidence-based medicine (EBM) is about the interaction of individual clinical expertise and the best available external evidence from a literature search. Sackett et al. define it as the conscientious, explicit, and judicious use of current best evidence in making decisions about the care of individual patients [15]. The terms EBM and EBP are sometimes used interchangeably, but in reality, whether it is EBM, evidence-based nursing, or EBLM, they all refer to the basic concept of EBP: the use of the best available evidence, coupled with a practitioner’s clinical proficiency and a patient’s preference, to inform the care of patients. EBM does not emphasize intuitive ability, unmethodical clinical experience, or pathological basis as sufficient grounds for clinical decision-making; rather, it emphasizes the examination of evidence from clinical research [16]. Price stated that EBM embraces diagnostic modalities, whereas EBLM focuses on the use of diagnostic tests and the goal of improving patient outcomes [17].

However, EBLM is about constructing clinical advice and management decisions based on the best available external information. The benefits and application of EBLM occur at many levels, and as an independent professional, you may have to deal with suggestions for new tests to be added to your laboratory test collection, as well as be prepared to be consulted on the usefulness of existing tests in relation to specific patients or groups of patients [18]. The compilation and evaluation of evidence take time and effort, and so the basic steps in EBP include asking the right question, acquiring the evidence, appraising the evidence, applying the evidence, and assessing the experience (Ask, Acquire, Appraise, Apply, and Assess). The growth of conversion of these steps into daily clinical and laboratory practice has been very limited [17].

Laboratory tests are among the most commonly used diagnostic approaches to support medical decision-making, yet the evidence demonstrating the impact of laboratory testing on clinical outcomes is limited. Evidence-based guidelines are expected to bridge the gap between research and clinical practice, promoting the valuable use of laboratory medicine [19].

In addition, clinicians may depend on recommendations from clinical practice guidelines for patient management, which are published statements that include recommendations intended to achieve the best patient care. In this process, a group of experts formulates recommendation questions that guide the retrieval of information used as evidence to inform the best practice guidelines [20].

However, clinical decisions should rely on the totality of the best available evidence, not just the results of individual studies. Applying the results of a systematic review or meta-analysis to patient care should start with an evaluation of the methodology to understand the extent to which these methods are protected against error and misleading results [21]. The addition of laboratory practice to EBM was a step in the right direction, as medical decisions include those based on laboratory test results, which comprise approximately 70% of healthcare decisions affecting diagnosis or treatment that involve laboratory investigations [16,22]. Thus, the total testing process (TTP), including laboratory and clinicians’ requests for tests, could introduce errors that may contribute to adverse health outcomes and reduce the quality of care and patient satisfaction. The most common of these errors include an incorrect sequence of tests, incorrect patient and specimen identification, improperly performed lab tests, incorrect result interpretation, and incorrect results verification.

Consequent upon these, there was a need for evidence-based models for recommending laboratory medicine practices, and the CDC’s Division of Laboratory System (DLS) led a concerted national effort to apply an evidence-based approach to evaluating and recommending best practices in laboratory medicine, in line with the Institute of Medicine (IOM) recommendations [23].

Laboratory Testing Algorithms

A laboratory testing algorithm is a standardized, step-by-step, guideline-informed process for selecting, performing, and interpreting a sequence of laboratory tests to achieve an efficient diagnosis. Laboratory diagnostic pathways combine step-by-step reflex testing with a cost advantage and are constructed based on an expert guide, which can be seen as a decision tree. In daily clinical practice, the diagnostic pathway can be viewed as a quick test outline that guides clinicians in making decisions effectively and efficiently [24].

Testing algorithms are designed to help healthcare professionals make informed decisions about which tests to select and perform, with an interpretive guide to results reflecting the patient’s symptoms, medical history, epidemiology, and risk factors. These algorithms usually display a sequence of tests performed in a specific order for optimal diagnostic accuracy and efficiency [25]. Reflex testing algorithms, coupled with a narrative interpretive guide provided by laboratory professionals, help providers arrive at a definitive diagnosis without ordering individual tests [26].

Consequently, these strategic approaches help make the process easier, save costs, and reduce the number of false-positive and false-negative results [24]. In the field of laboratory medicine, reducing errors and creating standardization are only possible through a predefined process; hence, the need for a decision-algorithm model that can efficiently and rapidly evaluate laboratory test results. Demirci et al. developed an artificial neural network approach for laboratory test reporting, designed to be integrated into laboratory information systems (LIS) to improve efficiency and quality of work, while still maintaining patient safety [27]. Moreover, machine learning algorithms can be used to authenticate the decision tree established by a multidisciplinary team (MDT) or to suggest potential new trees where guidelines are not readily available [24]. It is crucial to understand that testing algorithms are dynamic and are regularly updated as new evidence emerges and diagnostic technologies advance. As a result, healthcare professionals must stay abreast of the latest guidelines and put them into practice promptly. Furthermore, it is difficult for healthcare professionals who lack guidance to keep up with scientific advances and changing practice guidelines relevant to ordering and interpreting laboratory results [28].

For this reason, interpretation can improve a physician’s ability to select the appropriate test needed to determine or exclude a diagnosis. Interpretive services are very efficient when combined with strategic testing algorithms that simplify the diagnostic process for healthcare professionals [29]. The question to ask is: what is reflex testing, and how is it different from first-line testing? Reflex testing is an auto-generated secondary test that is triggered when the result of a first-line test meets certain predefined criteria. It is faster and more efficient because it eliminates the need for a separate test request. The implementation of reflex testing reduces the time frame from diagnosis to the delivery of results, while also creating capacity for more patients to be tested [30]. Thus, the difference between a first-line test (the initial test performed to screen or diagnose) and reflex testing lies in efficiency and purpose.

Finally, sources of evidence for laboratory testing algorithms include clinical studies, systematic reviews, meta-analyses, and guideline recommendations from an MDT.

Benefits of evidence-based testing algorithms

One of the greatest benefits of evidence-based algorithms is the reduction of unnecessary tests. Laboratory test overuse is very common in healthcare systems, and unnecessary phlebotomy can lead to the wastage of resources and may cause iatrogenic anemia in patients [31]. Secondly, evidence-based testing algorithms are a construct of the laboratory professional’s role that improves diagnostic accuracy and ultimately enhances the quality of healthcare. Most physicians request an interpretive guide for laboratory evaluations that contain abnormal test results, and thus, the use of algorithms helps avoid misdiagnosis by reducing errors and ensuring comprehensive testing [26].

Thirdly, the use of laboratory testing algorithms has been shown to promote cost-effectiveness; thus, algorithmic testing has contributed to reducing laboratory test ordering without compromising the quality of care, even as laboratory costs drive many medical decisions [11]. The cost of excessive laboratory testing includes the additional costs of performing tests and an increased likelihood of costs arising from false-positive results. The display of the probability of obtaining abnormal results, along with computer-aided reminders of clearly unnecessary tests, has been shown to be effective in reducing the rate of unnecessary test ordering by physicians, thereby lowering costs [32]. Healthcare budgets in every nation are facing increasing pressure to reduce costs while maintaining quality of care [33]; hence, embracing the application of testing algorithms can have a positive impact on providing cost-effective care.

Finally, the creation of systematic, algorithmic approaches to improving the diagnostic process in healthcare has helped reduce the hindrances to the speed and accuracy of diagnosis caused by the volume and complexity of tests, thereby providing enhanced clinical decision support [34].

Common scenario of laboratory testing algorithm in clinical setting

**Figure 1:**
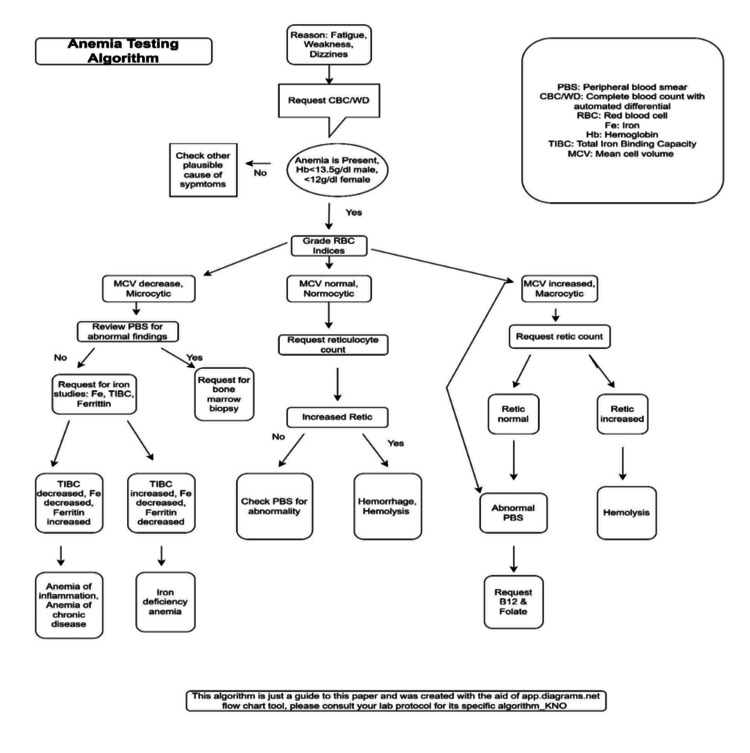
A typical anemia testing algorithm created using apps.diagrams.net flow chart tool

Repetitive laboratory testing and over-ordering, with their attendant issues, triggered an intervention championed by many medical societies, especially the initiative led by the American Board of Internal Medicine Foundation in 2013, promoting the Choosing Wisely recommendations to minimize redundant daily laboratory tests by providing education to physicians and patients on areas of test overuse [35,36]. In this regard, we describe the application and benefits of laboratory testing algorithms, using anemia testing algorithms as a common scenario frequently encountered in clinical settings. Evaluation for anemia is a common problem in clinical practice, and while it may appear straightforward in healthy individuals with a single cause, it can be overwhelming when multiple conditions contribute to the problem [37]. A good example is presented in Figure 1, which provides an algorithmic framework portraying testing and classification pathways for anemia diagnosis as follows in the clinical setting. This diagnostic framework guides a sequential protocol for testing, analyzing, and classifying anemia. It also provides interpretive guidance, and when embedded in communication between the electronic health record (EHR) and LIS, it can offer a comprehensive interpretive guide for making accurate diagnoses and improving data management. The benefits of this application include enhanced efficiency, reduced provider workload, lower costs of care, and faster diagnosis by increasing the likelihood of optimal test selection. Machine learning and artificial intelligence are making their way to improving this process as laboratory medicine advances.

A classic example of an established algorithmic pathway for the differential diagnosis of anemia is shown. The pathway usually starts with a complete blood count, with a focus on hemoglobin values and RBC indices, especially mean corpuscular volume (MCV). When anemia is not present, other plausible causes of symptoms can be evaluated. If anemia is present, with low hemoglobin values, the algorithm provides a step-by-step path to follow to reach a definitive diagnosis.

Challenges in the implementation of laboratory test algorithms

The EBLM approach to improving diagnostic accuracy is an area of medicine that is currently receiving attention; yet, there are a few limitations that have hindered attempts to popularize the approach through the expertise of laboratory professionals. In the process of preparing this paper, it was discovered that there is limited material evidence to support laboratory medicine, even though there are many systematic reviews that address the problem without directly tackling its core issues. This challenge is attributed to the difficulty of generating evidence to demonstrate that laboratory medicine can contribute to bridging the existing gap and making the best use of the effectiveness of routine and clinical services already in place, because it is evidence that ultimately makes the case [17].

Another challenge to the implementation of testing algorithms is the lack of full integration with the EHR. Thus, the three components of the EHR that must communicate with the LIS are the clinical-decision support system (CDSS), the computerized physician order entry (CPOE) system, and the health information exchange (HIE) system. The CDSS is a system designed to help providers make specific decisions for each patient based on information contained in the EHR and clinical guidelines embedded within the system [2]. As a result of this lack of full integration, the EHR may be an underutilized resource for reducing unnecessary laboratory tests [35], as algorithm integration would have made it more efficient.

Variability in practice is also a challenge to the implementation of testing algorithms in a standardized form across healthcare organizations. Part of the solution may be provided by an HIE system that enables access to information among healthcare providers using nationally recognized standards. In addition, HIE can help maximize the usability of CPOE by making available to providers all tests, along with their clinical utility, across different laboratories or healthcare organizations [2]. There has also been mention of a program developed within the Southern California Kaiser Permanente healthcare system, called SureNet, which uses a tracking and alert system based on clinical laboratory data abstracted from patient electronic records [13].

Need for collaboration among healthcare professionals to deliver the best of care

The laboratory professionals have a unique position to lead the development and implementation of testing algorithms. Clinical research in laboratory medicine emphasizes diagnostic test accuracy, precision, value, and results. Therefore, because of advances in methods and technologies, such laboratory evidence often refutes previously accepted diagnostic tests and replaces them with the most current findings, which help to achieve better patient outcomes more efficiently and effectively, with cost in view, thus promoting the value of the role of laboratory professionals [22]. The role of laboratory professionals in providing advice on laboratory test selection and interpretation [26] is useful for understanding the interface between the clinical team and laboratory staff, where both meet at the intersection to work together for better-quality patient care [38].

Effective communication and collaboration between laboratory and clinical teams is crucial for the delivery of the best care to patients and for supporting the continued advances in laboratory medicine. These advances will help to build better strategies for disease prevention and diagnosis [39]. Enhanced communication is also vitally important in fostering participation, which entails providing educational information and assistance [40]. Moreover, inter-professional experience and education serve as an engaging platform for increasing awareness of the unique role of laboratory professionals in healthcare. Collaborative and active engagement of professionals from different healthcare disciplines helps bring laboratory professionals to the stage to present their expertise and gain recognition in overall patient care activities [41].

The clinical laboratory tests are increasing in number and complexity, so an understanding of their clinical utility is crucial to supporting accurate and timely diagnosis [13], thus necessitating effective collaboration to eradicate process waste, improve the speed of diagnosis, and achieve better patient outcomes.

## Conclusions

A diagnostic approach without laboratory medicine input is like groping in the daytime as in the thickness of a dark night, and that is why the intention of this paper is to throw strong illumination on the role of laboratory professionals in using an evidence-based laboratory testing algorithm to fine-tune diagnostic accuracy. The pursuit to reduce errors, reduce over-utilization of laboratory tests, amend underuse, and reduce healthcare costs without compromising the quality of care is all embedded in the TTP, which directly or indirectly involves multidisciplinary input to achieve greater diagnostic efficiency and improved patient treatment outcomes.

The value of collaboration between laboratory professionals and clinical teams will drive the future in shaping diagnostic strategies and calls for an expanded awareness through education, familiarity, and the full integration of EBLM in diagnostic approaches, to provide solutions with better outcomes.

## References

[1] Price CP, Christenson RH (2013). Ask the right question: a critical step for practicing evidence-based laboratory medicine. Ann Clin Biochem.

[2] Aziz HA, Alshekhabobakr HM (2017). Health informatics tools to improve utilization of laboratory tests. Lab Med.

[3] Lippi G, Cervellin G (2017). From laboratory instrumentation to physician’s brain calibration: the next frontier for improving diagnostic accuracy?. J Lab Precis Med.

[4] Balogh EP, Miller BT, Ball JR (2015). The diagnostic process. Improving Diagnosis in Healthcare.

[5] Alharbi TA, Rababa M, Alsuwayl H, Alsubail A, Alenizi WS (2025). Diagnostic challenges and patient safety: the critical role of accuracy - a systematic review. J Multidiscip Healthc.

[6] Olson AP, Graber ML (2020). Improving diagnosis through education. Acad Med.

[7] Centor RM, Geha R, Manesh R (2019). The pursuit of diagnostic excellence. JAMA Netw Open.

[8] Graber ML (2008). Taking steps towards a safer future: measures to promote timely and accurate medical diagnosis. Am J Med.

[9] Šimundić AM (2009). Measures of diagnostic accuracy: basic definitions. EJIFCC.

[10] Carrigan I, Ma IW, Ambasta A (2022). A framework for purposeful utilization of laboratory tests in hospitalized patients. Am J Med.

[11] Shaik T, Mahmood R, Kanagala SG (2024). Lab testing overload: a comprehensive analysis of overutilization in hospital-based settings. Proc (Bayl Univ Med Cent).

[12] Conroy M, Homsy E, Johns J (2021). Reducing unnecessary laboratory utilization in the medical ICU: a fellow-driven quality improvement initiative. Crit Care Explor.

[13] Lubin IM, Astles JR, Shahangian S, Madison B, Parry R, Schmidt RL, Rubinstein ML (2021). Bringing the clinical laboratory into the strategy to advance diagnostic excellence. Diagnosis (Berl).

[14] Shreffler J, Huecker MR (2023). Diagnostic testing accuracy: sensitivity, specificity, predictive values and likelihood ratios. StatPearls [Internet].

[15] Sackett DL, Rosenberg WM, Gray JA, Haynes RB, Richardson WS (1996). Evidence based medicine: what it is and what it isn't. BMJ.

[16] Badrick T (2013). Evidence-based laboratory medicine. Clin Biochem Rev.

[17] Price CP (2012). Evidence-based laboratory medicine: is it working in practice?. Clin Biochem Rev.

[18] Whitefield JB (2013). Evidence based laboratory medicine for beginners: why evidence matters. Chem Pathol.

[19] Whitfield JB (2013). Evidence based laboratory medicine for beginners. Pathology.

[20] Brignardello-Petersen R, Carrasco-Labra A, Guyatt GH (2021). How to interpret and use a clinical practice guideline or recommendation: users' guides to the medical literature. JAMA.

[21] Murad MH, Montori VM, Ioannidis JP (2014). How to read a systematic review and meta-analysis and apply the results to patient care: users' guides to the medical literature. JAMA.

[22] Weissfeld AS, Baselski V, Cornish NE (2024). The American Society for Microbiology collaboration with the CDC Laboratory Medicine Best Practices initiative for evidence-based laboratory medicine. Clin Microbiol Rev.

[23] Centers for Disease Control and Prevention (2008). Laboratory Medicine Best Practices: Developing an Evidence-Based Review and Evaluation Process. Final Technical Report 2007: Phase I. https://stacks.cdc.gov/view/cdc/44238/cdc_44238_DS1.pdf.

[24] Hoffmann G, Bietenbeck A, Lichtinghagen R (2018). Using machine learning techniques to generate laboratory diagnostic pathways - a case study. J Lab Precis Med.

[25] Roche Diagnostics. (2024, March 14 (2025). The crucial role of testing algorithms in the diagnosis of infectious diseases. https://diagnostics.roche.com/global/en/article-listing/health-topics/infectious-disease/testing-algorithms-for-diagnosing-infectious-diseases.html.

[26] Kratz A, Laposata M (2002). Enhanced clinical consulting - moving toward the core competencies of laboratory professionals. Clinica Chimica Acta.

[27] Demirci F, Akan P, Kume T, Sisman AR, Erbayraktar Z, Sevinc S (2016). Artificial neural network approach in laboratory test reporting: learning algorithms. Am J Clin Pathol.

[28] Jackson BR (2007). Managing laboratory test use: principles and tools. Clin Lab Med.

[29] Van Cott EM (2014). Laboratory test interpretations and algorithms in utilization management. Clin Chim Acta.

[30] Gosney JR, Paz-Ares L, Jänne P (2023). Pathologist-initiated reflex testing for biomarkers in non-small-cell lung cancer: expert consensus on the rationale and considerations for implementation. ESMO Open.

[31] Huang T, Li LT, Bernstam EV, Jiang X (2023). Confidence-based laboratory test reduction recommendation algorithm. BMC Med Inform Decis Mak.

[32] Schubart JR, Fowler CE, Donowitz GR (2001). Algorithm-based decision rules to safely reduce laboratory test ordering. Stud Health Technol Inform.

[33] Medidani Z, Farzandipour M, Farrokhian A (2016). A review on laboratory tests’ utilization: A trigger for cutting costs and quality improvement in healthcare settings. Med J Islam Repub Iran.

[34] Laposata ME, Laposata M, Van Cott EM, Buchner DS, Kashalo MS, Dighe AS (2004). Physician survey of a laboratory medicine interpretive service and evaluation of the influence of interpretations on laboratory test ordering. Arch Pathol Lab Med.

[35] Parsons AS, Wiencek JR (2019). The ABCs of Reducing Repetitive Laboratory Testing. TODAY’S CLINICAL LAB, https://www.clinicallab.com/.

[36] Yeshoua B, Bowman C, Dullea J (2023). Interventions to reduce repetitive ordering of low-value inpatient laboratory tests: a systematic review. BMJ Open Qual.

[37] Means Means, R. T., & Brodsky, R. A. (2025 (2025). Diagnostic approach to anemia in adults. https://www.uptodate.com/contents/diagnostic-approach-to-anemia-in-adults.

[38] van den Broek A, Tuijn CJ, van't Klooster L, Msoka E, Boer MS, Chilongola J, Oskam L (2014). Understanding the interface between clinical and laboratory staff. Afr J Lab Med.

[39] Dabla PK, Sethi HK (2025). Enhancing clinical collaboration: Interface linking between laboratories and clinics for translational healthcare. J Diabetes Clin Res.

[40] Fares Abanami HA, Hazzazy AS, Al-Mutairi AS (2022). From diagnosis to treatment: the collaborative efforts of laboratory, nursing, medical devices, pharmacy technician in patient management. J Popul Ther Clin Pharmacol.

[41] Vu T (2024). The importance of inter-professional education for medical laboratory professionals. Am Soc Clin Lab Sci.

